# Supplementary material for: Conditional chemoconnectomics (cCCTomics) as a strategy for efficient and conditional targeting of chemical transmission
Source: eLife. 2024 Apr 30;12:RP91927. doi: 10.7554/eLife.91927 (PMC11060718; doi:10.7554/eLife.91927)
Supplement: Figure 3—source data 3. [file elife-91927-fig3-data3.zip › Figure 3 source data3 part 1.zip!/Figure 3-Source data 3-part1/1063f3-1-CRISPResso_Report_kboyr13q.zip!/CRISPResso_on_1063f3_2/2a.Nucleotide_percentage_quilt.pdf]

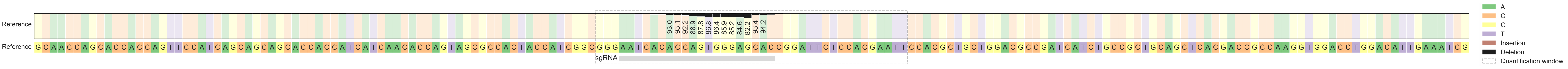

Reference
Reference
GCAACCAGCACCACCAGTTCCATCAGCAGCAGCACCACCATCATCAACACCAGTAGCGCCACTACCATCGGCGGGAATCACACCAGTGGGAGCACCGGATTCTCCACGAATTCCACGCTGCTGGACGCCGATCATCTGCCGCTGCAGCTCACGACCGCCAAGGTGGACCTGGACATTGAAATCG
93.0
93.1
92.2
88.9
87.8
86.8
86.4
85.9
85.2
84.6
82.2
93.4
94.2
sgRNA
A
C
G
T
Insertion
Deletion
Quantification window
